# (*E*)-1-(4-Chloro­phen­yl)-3-[4-(2,3,4,6-tetra-*O*-acetyl-β-d-allopyranos­yloxy)phen­yl]prop-2-en-1-one

**DOI:** 10.1107/S1600536809055652

**Published:** 2010-01-09

**Authors:** Kuan Zhang, Xue Bai, Hua-Feng Chen, Ying Li, Shu-Fan Yin

**Affiliations:** a College of Chemistry, Sichuan University, Chengdu 610064, People’s Republic of China

## Abstract

The asymmetric unit of the title compound, C_29_H_29_ClO_11_, contains two independent mol­ecules of similar geometry, both adopting an *E* conformation about the C=C double bond. The dihedral angles formed by benzene rings are 10.73 (16) and 13.79 (18)°. The pyran­oside rings adopt a chair conformation. Intra­molecular C—H⋯O close contacts occur. The crystal packing is stabilized by inter­molecular C—H⋯O hydrogen bonds.

## Related literature

For the biological properties of helicid [systematic name: 4-formyl­phenl-β-D-allopyran­oside], see: Chen *et al.* (1981 ▶); Sha & Mao (1987 ▶). For the crystal structures of helicid derivatives, see: Fan *et al.* (2007 ▶); Fu *et al.* (2009 ▶); Ye *et al.* (2009 ▶): Yang *et al.* (2009 ▶).
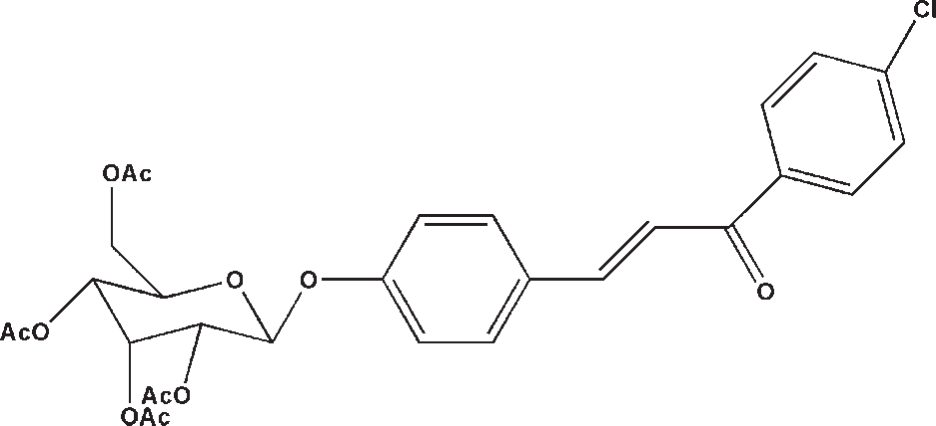


## Experimental

### 

#### Crystal data

C_29_H_29_ClO_11_
*M* *_r_* = 588.97
Monoclinic, 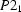

*a* = 7.2387 (14) Å
*b* = 39.239 (8) Å
*c* = 10.297 (2) Å
β = 90.25 (3)°
*V* = 2924.7 (10) Å^3^
*Z* = 4
Mo *K*α radiation
μ = 0.19 mm^−1^
*T* = 113 K
0.27 × 0.25 × 0.20 mm

#### Data collection

Rigaku Saturn CCD area-detector diffractometer
Absorption correction: multi-scan (*CrystalClear*; Rigaku/MSC, 2005 ▶) *T*_min_ = 0.951, *T*_max_ = 0.963
17131 measured reflections
11143 independent reflections
8341 reflections with *I* > 2σ(*I*)
*R*_int_ = 0.041

#### Refinement

*R*[*F* ^2^ > 2σ(*F* ^2^)] = 0.067
*wR*(*F* ^2^) = 0.189
*S* = 1.04
11143 reflections
748 parameters
1 restraint
H-atom parameters constrained
Δρ_max_ = 0.76 e Å^−3^
Δρ_min_ = −0.37 e Å^−3^
Absolute structure: Flack (1983 ▶); 4380 Friedel pairs
Flack parameter: 0.03 (8)

### 

Data collection: *CrystalClear* (Rigaku/MSC, 2005 ▶); cell refinement: *CrystalClear*; data reduction: *CrystalClear*; program(s) used to solve structure: *SHELXS97* (Sheldrick, 2008 ▶); program(s) used to refine structure: *SHELXL97* (Sheldrick, 2008 ▶); molecular graphics: *ORTEP-3 for Windows* (Farrugia, 1997 ▶); software used to prepare material for publication: *SHELXL97*.

## Supplementary Material

Crystal structure: contains datablocks global, I. DOI: 10.1107/S1600536809055652/rz2404sup1.cif

Structure factors: contains datablocks I. DOI: 10.1107/S1600536809055652/rz2404Isup2.hkl

Additional supplementary materials:  crystallographic information; 3D view; checkCIF report

## Figures and Tables

**Table 1:** Hydrogen-bond geometry (Å, °)

*D*—H⋯*A*	*D*—H	H⋯*A*	*D*⋯*A*	*D*—H⋯*A*
C1—H1⋯O9^i^	1.00	2.38	3.185 (5)	137
C20—H20⋯O1	0.95	2.54	3.111 (5)	118
C20—H20⋯O9^i^	0.95	2.54	3.271 (5)	134
C30—H30⋯O20^i^	1.00	2.51	3.316 (5)	138
C32—H32⋯O20	1.00	2.53	3.031 (5)	111
C49—H49⋯O12	0.95	2.50	3.065 (5)	119
C49—H49⋯O20^i^	0.95	2.53	3.271 (6)	135

Symmetry code: (i) 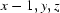.

**Fig. 1.**
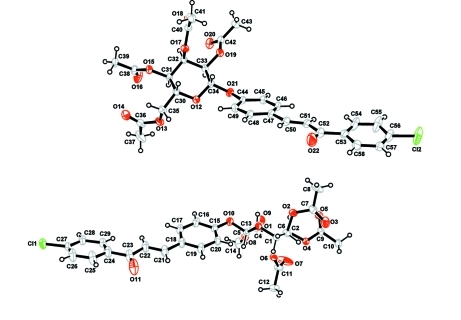
The asymmetric unit of the title compound, with displacement ellipsoids drawn at the 30% probability level.

## supplementary crystallographic information

### Comment

The natural compound hecilid [systematic name: 4-formylphenl-β-D-allopyranoside], C_13_H_16_O_7_, extracted from the fruit of Helicia Nilagirica Beed (Chen *et al.*, 1981), is a major active ingredient of Chinese herbal medicine. It has been reported to possess a variety of biological effects on the central nervous system including sedative, hypnotic and anticonvulsant activities (Sha & Mao, 1987). In order to improve its hypnotic-sedative activity, we have recently synthesized helicid derivatives and reported their structures (Fan *et al.*, 2007; Fu *et al.*, 2009); Ye *et al.*, 2009, Yang *et al.*, 2009).

The title compound crystallizes with two independent molecules in the asymmetric unit (Fig. 1), adopting an *E* conformation about the C21═C22 and C50═C51 double bonds. The average of C–C bond length in the pyranoside rings is 1.520 (5) Å, the average C(*sp*^3^)–O and C(*sp*^2^)–O bond lengths are 1.436 (4) and 1.360 (5) Å, respectively. The pyranoside rings adopt the stable chair conformation. The substituents of the protected sugar at the C3 are in the axial position, while all other groups are in equatorial positions. The dihedral angles formed by the benzene rings in the two molecules are 10.73 (16) and 13.79 (18)°, respectively. The crystal packing is stabilized by intra- and intermolecular C—H···O hydrogen bonds (Table 1).

### Experimental

To a solution of E-4-β-D-allopyranoside-cinnamic-4-chlorophenyl ketone (2.06 g, 3.5 mmol) in DMF (2 ml) and triethanolamine (3 ml) was added dropwise acetyl anhydride (2.5 g, 25 mmol) under ice bath. The mixture was stirred vigorously at room temperature  for5 h, then poured into 20 ml of ice water. The precipitate was filtered and washed with water. Colourless crystals suitable for X-ray analysis were obtained by slow evaporation of an ethanol solution.

### Refinement

All H atoms were positioned geometrically and refined using a riding model, with C—H = 0.95–1.00 Å, and with  *U*_iso_(H) = 1.2 *U*_eq_ (C) or 1.5 *U*_eq_ (C) for methyl H atoms.

### Crystal data

**Table:**

C_29_H_29_ClO_11_	*F*(000) = 1232
*M**_r_* = 588.97	*D*_x_ = 1.338 Mg m^−^^3^
Monoclinic, *P*2_1_	Mo *K*α radiation, λ = 0.71073 Å
Hall symbol: P 2yb	Cell parameters from 5501 reflections
*a* = 7.2387 (14) Å	θ = 2.0–27.9°
*b* = 39.239 (8) Å	µ = 0.19 mm^−^^1^
*c* = 10.297 (2) Å	*T* = 113 K
β = 90.25 (3)°	Block, colourless
*V* = 2924.7 (10) Å^3^	0.27 × 0.25 × 0.20 mm
*Z* = 4	

### Data collection

**Table:**

Rigaku Saturn CCD area-detector diffractometer	11143 independent reflections
Radiation source: rotating anode	8341 reflections with *I* > 2σ(*I*)
confocal	*R*_int_ = 0.041
Detector resolution: 7.31 pixels mm^-1^	θ_max_ = 27.9°, θ_min_ = 2.0°
ω and φ scans	*h* = −9→6
Absorption correction: multi-scan (*CrystalClear*; Rigaku/MSC, 2005)	*k* = −49→51
*T*_min_ = 0.951, *T*_max_ = 0.963	*l* = −13→13
17131 measured reflections	

### Refinement

**Table:**

Refinement on *F*^2^	Hydrogen site location: inferred from neighbouring sites
Least-squares matrix: full	H-atom parameters constrained
*R*[*F*^2^ > 2σ(*F*^2^)] = 0.067	*w* = 1/[σ^2^(*F*_o_^2^) + (0.0899*P*)^2^] where *P* = (*F*_o_^2^ + 2*F*_c_^2^)/3
*wR*(*F*^2^) = 0.189	(Δ/σ)_max_ < 0.001
*S* = 1.03	Δρ_max_ = 0.76 e Å^−^^3^
11143 reflections	Δρ_min_ = −0.37 e Å^−^^3^
748 parameters	Extinction correction: *SHELXL97* (Sheldrick, 2008)
1 restraint	Extinction coefficient: 0.0394 (19)
Primary atom site location: structure-invariant direct methods	Absolute structure: Flack (1983); 4380 Friedel pairs
Secondary atom site location: difference Fourier map	Flack parameter: 0.03 (8)

### Special details

**Table:**

Geometry. All e.s.d.'s (except the e.s.d. in the dihedral angle between two l.s. planes) are estimated using the full covariance matrix. The cell e.s.d.'s are taken into account individually in the estimation of e.s.d.'s in distances, angles and torsion angles; correlations between e.s.d.'s in cell parameters are only used when they are defined by crystal symmetry. An approximate (isotropic) treatment of cell e.s.d.'s is used for estimating e.s.d.'s involving l.s. planes.
Refinement. Refinement of *F*^2^ against ALL reflections. The weighted *R*-factor *wR* and goodness of fit *S* are based on *F*^2^, conventional *R*-factors *R* are based on *F*, with *F* set to zero for negative *F*^2^. The threshold expression of *F*^2^ > 2σ(*F*^2^) is used only for calculating *R*-factors(gt) *etc*. and is not relevant to the choice of reflections for refinement. *R*-factors based on *F*^2^ are statistically about twice as large as those based on *F*, and *R*- factors based on ALL data will be even larger.

### Fractional atomic coordinates and isotropic or equivalent isotropic displacement parameters (Å^2^)

**Table:**

	*x*	*y*	*z*	*U*_iso_*/*U*_eq_	
Cl1	−0.4045 (2)	0.46856 (4)	−0.80542 (16)	0.0871 (6)	
O1	0.0935 (4)	0.23162 (7)	0.2486 (2)	0.0331 (6)	
O2	0.0223 (4)	0.22091 (8)	0.5168 (3)	0.0409 (7)	
O3	−0.0068 (5)	0.17591 (10)	0.6492 (3)	0.0583 (10)	
O4	0.1770 (4)	0.14634 (7)	0.3739 (2)	0.0325 (6)	
O5	0.3944 (5)	0.15196 (10)	0.5296 (4)	0.0676 (11)	
O6	0.2152 (3)	0.15628 (7)	0.1075 (2)	0.0307 (6)	
O7	0.4675 (4)	0.13160 (8)	0.0196 (3)	0.0467 (8)	
O8	0.4323 (3)	0.20767 (7)	0.0084 (2)	0.0327 (6)	
O9	0.7123 (4)	0.19670 (8)	0.0942 (3)	0.0454 (8)	
O10	0.2147 (4)	0.26256 (7)	0.0803 (3)	0.0361 (6)	
O11	−0.6370 (8)	0.35722 (14)	−0.3468 (4)	0.0944 (16)	
C1	0.0449 (5)	0.19838 (10)	0.2976 (3)	0.0327 (8)	
H1	−0.0276	0.1859	0.2297	0.039*	
C2	0.2234 (5)	0.17956 (10)	0.3222 (3)	0.0296 (8)	
H2	0.3002	0.1924	0.3867	0.036*	
C3	0.3300 (5)	0.17529 (10)	0.1964 (3)	0.0301 (8)	
H3	0.4502	0.1634	0.2120	0.036*	
C4	0.3608 (5)	0.21054 (10)	0.1381 (3)	0.0299 (8)	
H4	0.4477	0.2240	0.1938	0.036*	
C5	0.1765 (5)	0.22917 (10)	0.1247 (3)	0.0303 (8)	
H5	0.0931	0.2170	0.0625	0.036*	
C6	−0.0756 (6)	0.20335 (12)	0.4162 (4)	0.0375 (9)	
H6A	−0.1868	0.2166	0.3916	0.045*	
H6B	−0.1166	0.1809	0.4487	0.045*	
C7	0.0449 (6)	0.20507 (14)	0.6306 (4)	0.0431 (10)	
C8	0.1309 (8)	0.22760 (18)	0.7274 (4)	0.0663 (16)	
H8A	0.0371	0.2428	0.7638	0.099*	
H8B	0.2276	0.2412	0.6858	0.099*	
H8C	0.1855	0.2138	0.7971	0.099*	
C9	0.2725 (6)	0.13525 (11)	0.4806 (4)	0.0394 (9)	
C10	0.2024 (6)	0.10208 (11)	0.5231 (4)	0.0405 (10)	
H10A	0.2790	0.0935	0.5947	0.061*	
H10B	0.2066	0.0860	0.4505	0.061*	
H10C	0.0745	0.1046	0.5526	0.061*	
C11	0.3029 (6)	0.13577 (10)	0.0206 (4)	0.0366 (9)	
C12	0.1689 (7)	0.12083 (13)	−0.0733 (4)	0.0469 (11)	
H12A	0.1177	0.1389	−0.1282	0.070*	
H12B	0.0687	0.1097	−0.0258	0.070*	
H12C	0.2319	0.1040	−0.1278	0.070*	
C13	0.6163 (5)	0.19973 (11)	−0.0015 (4)	0.0380 (9)	
C14	0.6735 (7)	0.19570 (13)	−0.1389 (4)	0.0514 (12)	
H14A	0.7064	0.2180	−0.1748	0.077*	
H14B	0.5713	0.1859	−0.1893	0.077*	
H14C	0.7807	0.1805	−0.1434	0.077*	
C15	0.0739 (6)	0.27878 (10)	0.0115 (4)	0.0364 (9)	
C16	0.1302 (7)	0.30255 (11)	−0.0791 (4)	0.0427 (10)	
H16	0.2573	0.3080	−0.0883	0.051*	
C17	−0.0025 (8)	0.31829 (12)	−0.1565 (4)	0.0526 (13)	
H17	0.0352	0.3346	−0.2194	0.063*	
C18	−0.1872 (8)	0.31079 (12)	−0.1440 (4)	0.0472 (11)	
C19	−0.2401 (7)	0.28779 (12)	−0.0488 (4)	0.0442 (10)	
H19	−0.3677	0.2830	−0.0377	0.053*	
C20	−0.1119 (6)	0.27163 (11)	0.0308 (4)	0.0396 (9)	
H20	−0.1501	0.2562	0.0963	0.047*	
C21	−0.3332 (8)	0.32739 (14)	−0.2210 (5)	0.0579 (14)	
H21	−0.4555	0.3196	−0.2065	0.069*	
C22	−0.3144 (9)	0.35191 (13)	−0.3078 (4)	0.0595 (15)	
H22	−0.1949	0.3605	−0.3273	0.071*	
C23	−0.4788 (10)	0.36618 (16)	−0.3751 (5)	0.0662 (16)	
C24	−0.4528 (9)	0.39271 (13)	−0.4791 (4)	0.0576 (15)	
C25	−0.6059 (10)	0.40906 (17)	−0.5284 (5)	0.080 (2)	
H25	−0.7238	0.4039	−0.4933	0.096*	
C26	−0.5936 (10)	0.43274 (15)	−0.6271 (5)	0.0699 (18)	
H26	−0.7010	0.4437	−0.6599	0.084*	
C27	−0.4251 (10)	0.43989 (13)	−0.6758 (5)	0.0652 (17)	
C28	−0.2635 (10)	0.42421 (14)	−0.6317 (6)	0.080 (2)	
H28	−0.1468	0.4290	−0.6693	0.096*	
C29	−0.2821 (10)	0.40076 (13)	−0.5282 (5)	0.0651 (17)	
H29	−0.1750	0.3904	−0.4921	0.078*	
Cl2	−0.4194 (3)	0.24933 (4)	1.58590 (17)	0.0931 (7)	
O12	0.1131 (4)	0.48458 (7)	0.5432 (2)	0.0332 (6)	
O13	0.0396 (4)	0.49401 (8)	0.2686 (2)	0.0383 (7)	
O14	0.0283 (5)	0.54079 (9)	0.1405 (3)	0.0531 (9)	
O15	0.2201 (4)	0.56671 (7)	0.3966 (2)	0.0317 (6)	
O16	0.4724 (4)	0.55925 (9)	0.2730 (3)	0.0503 (8)	
O17	0.2470 (4)	0.56115 (7)	0.6643 (2)	0.0322 (6)	
O18	0.4752 (6)	0.59742 (12)	0.6962 (5)	0.1016 (19)	
O19	0.4507 (4)	0.51189 (7)	0.7829 (2)	0.0353 (6)	
O20	0.7336 (4)	0.52389 (9)	0.7032 (3)	0.0476 (8)	
O21	0.2323 (4)	0.45509 (7)	0.7148 (3)	0.0374 (6)	
O22	−0.6366 (6)	0.36340 (14)	1.1305 (4)	0.0853 (14)	
C30	0.0696 (5)	0.51697 (10)	0.4870 (3)	0.0306 (8)	
H30	0.0023	0.5310	0.5527	0.037*	
C31	0.2533 (5)	0.53414 (10)	0.4569 (3)	0.0320 (8)	
H31	0.3266	0.5194	0.3971	0.038*	
C32	0.3606 (5)	0.53960 (10)	0.5825 (3)	0.0285 (7)	
H32	0.4828	0.5505	0.5651	0.034*	
C33	0.3851 (5)	0.50576 (10)	0.6528 (3)	0.0333 (8)	
H33	0.4738	0.4909	0.6048	0.040*	
C34	0.1990 (6)	0.48786 (10)	0.6672 (3)	0.0318 (8)	
H34	0.1175	0.5008	0.7278	0.038*	
C35	−0.0543 (5)	0.51158 (11)	0.3716 (3)	0.0342 (8)	
H35A	−0.1636	0.4982	0.3983	0.041*	
H35B	−0.0983	0.5339	0.3394	0.041*	
C36	0.0665 (6)	0.51104 (13)	0.1566 (4)	0.0413 (10)	
C37	0.1418 (7)	0.48837 (17)	0.0542 (4)	0.0635 (16)	
H37A	0.0407	0.4754	0.0142	0.095*	
H37B	0.2311	0.4726	0.0930	0.095*	
H37C	0.2032	0.5022	−0.0121	0.095*	
C38	0.3431 (5)	0.57652 (11)	0.3034 (4)	0.0340 (8)	
C39	0.2970 (6)	0.61081 (12)	0.2540 (4)	0.0423 (10)	
H39A	0.3474	0.6281	0.3132	0.063*	
H39B	0.1625	0.6133	0.2486	0.063*	
H39C	0.3505	0.6138	0.1676	0.063*	
C40	0.3226 (6)	0.58947 (11)	0.7148 (4)	0.0420 (10)	
C41	0.1861 (6)	0.60711 (13)	0.8003 (4)	0.0465 (11)	
H41A	0.2385	0.6287	0.8308	0.070*	
H41B	0.1578	0.5926	0.8750	0.070*	
H41C	0.0725	0.6116	0.7510	0.070*	
C42	0.6307 (6)	0.52256 (11)	0.7942 (4)	0.0394 (9)	
C43	0.6705 (7)	0.53339 (16)	0.9307 (5)	0.0610 (14)	
H43A	0.7818	0.5476	0.9323	0.092*	
H43B	0.6902	0.5132	0.9850	0.092*	
H43C	0.5657	0.5465	0.9642	0.092*	
C44	0.0878 (6)	0.43935 (10)	0.7811 (4)	0.0356 (8)	
C45	0.1393 (7)	0.41400 (11)	0.8695 (4)	0.0434 (10)	
H45	0.2650	0.4075	0.8789	0.052*	
C46	0.0033 (7)	0.39862 (11)	0.9431 (4)	0.0446 (11)	
H46	0.0371	0.3812	1.0027	0.053*	
C47	−0.1812 (7)	0.40791 (12)	0.9324 (4)	0.0449 (11)	
C48	−0.2288 (6)	0.43279 (11)	0.8400 (4)	0.0419 (10)	
H48	−0.3546	0.4392	0.8299	0.050*	
C49	−0.0941 (6)	0.44838 (11)	0.7624 (4)	0.0393 (9)	
H49	−0.1276	0.4647	0.6985	0.047*	
C50	−0.3288 (8)	0.39263 (13)	1.0094 (4)	0.0514 (12)	
H50	−0.4492	0.4017	0.9962	0.062*	
C51	−0.3144 (8)	0.36731 (13)	1.0965 (4)	0.0542 (13)	
H51	−0.1964	0.3581	1.1169	0.065*	
C52	−0.4804 (9)	0.35371 (15)	1.1606 (5)	0.0604 (15)	
C53	−0.4584 (9)	0.32656 (13)	1.2633 (4)	0.0571 (14)	
C54	−0.2858 (9)	0.31860 (14)	1.3170 (5)	0.0609 (15)	
H54	−0.1778	0.3294	1.2849	0.073*	
C55	−0.2715 (10)	0.29441 (16)	1.4192 (6)	0.078 (2)	
H55	−0.1559	0.2891	1.4584	0.094*	
C56	−0.4352 (10)	0.27874 (13)	1.4595 (5)	0.0642 (17)	
C57	−0.6005 (10)	0.28634 (16)	1.4068 (5)	0.0691 (17)	
H57	−0.7080	0.2750	1.4368	0.083*	
C58	−0.6159 (9)	0.31037 (17)	1.3100 (5)	0.0726 (18)	
H58	−0.7339	0.3159	1.2750	0.087*	

### Atomic displacement parameters (Å^2^)

**Table:**

	*U*^11^	*U*^22^	*U*^33^	*U*^12^	*U*^13^	*U*^23^
Cl1	0.1132 (12)	0.0597 (9)	0.0879 (10)	−0.0278 (9)	−0.0615 (10)	0.0396 (8)
O1	0.0384 (14)	0.0309 (14)	0.0301 (12)	0.0033 (12)	0.0032 (11)	0.0014 (11)
O2	0.0488 (17)	0.0437 (18)	0.0304 (13)	−0.0014 (14)	0.0054 (12)	−0.0050 (12)
O3	0.081 (3)	0.059 (2)	0.0346 (15)	0.007 (2)	0.0053 (16)	0.0065 (16)
O4	0.0344 (13)	0.0341 (15)	0.0289 (12)	−0.0017 (12)	−0.0007 (11)	0.0060 (11)
O5	0.073 (2)	0.064 (2)	0.066 (2)	−0.024 (2)	−0.0376 (19)	0.0269 (19)
O6	0.0304 (13)	0.0330 (15)	0.0286 (12)	−0.0033 (11)	−0.0024 (10)	−0.0047 (10)
O7	0.0412 (17)	0.056 (2)	0.0426 (15)	0.0091 (15)	0.0042 (14)	−0.0118 (15)
O8	0.0322 (13)	0.0378 (16)	0.0280 (12)	−0.0002 (12)	0.0001 (11)	0.0035 (11)
O9	0.0278 (14)	0.0521 (19)	0.0562 (18)	−0.0024 (13)	−0.0023 (13)	0.0171 (15)
O10	0.0437 (15)	0.0267 (14)	0.0379 (14)	−0.0021 (12)	−0.0011 (12)	0.0022 (11)
O11	0.102 (4)	0.105 (4)	0.076 (3)	0.048 (3)	0.020 (3)	0.044 (3)
C1	0.0361 (19)	0.036 (2)	0.0256 (16)	0.0059 (17)	−0.0016 (15)	0.0052 (15)
C2	0.0283 (17)	0.035 (2)	0.0257 (15)	−0.0012 (15)	−0.0005 (14)	0.0038 (15)
C3	0.0343 (19)	0.031 (2)	0.0243 (15)	−0.0041 (16)	−0.0044 (14)	0.0014 (14)
C4	0.0310 (18)	0.032 (2)	0.0264 (16)	−0.0044 (15)	−0.0002 (14)	0.0020 (14)
C5	0.0366 (19)	0.0256 (18)	0.0289 (16)	−0.0001 (16)	0.0015 (15)	0.0035 (14)
C6	0.035 (2)	0.043 (2)	0.0335 (18)	−0.0012 (18)	0.0018 (16)	0.0008 (17)
C7	0.039 (2)	0.059 (3)	0.0311 (19)	0.005 (2)	0.0061 (17)	−0.0018 (19)
C8	0.065 (3)	0.098 (5)	0.037 (2)	−0.020 (3)	0.009 (2)	−0.007 (3)
C9	0.041 (2)	0.040 (2)	0.0370 (19)	0.0045 (18)	−0.0048 (18)	0.0115 (18)
C10	0.046 (2)	0.036 (2)	0.040 (2)	−0.0033 (19)	0.0032 (18)	0.0129 (18)
C11	0.052 (2)	0.028 (2)	0.0293 (17)	−0.0035 (18)	0.0013 (17)	−0.0008 (15)
C12	0.055 (3)	0.051 (3)	0.035 (2)	−0.011 (2)	−0.0038 (19)	−0.0076 (19)
C13	0.034 (2)	0.035 (2)	0.045 (2)	0.0003 (17)	0.0016 (18)	0.0110 (18)
C14	0.058 (3)	0.046 (3)	0.050 (2)	0.007 (2)	0.022 (2)	0.005 (2)
C15	0.050 (2)	0.027 (2)	0.0315 (18)	0.0056 (18)	−0.0008 (17)	0.0050 (15)
C16	0.056 (3)	0.033 (2)	0.038 (2)	0.001 (2)	0.0101 (19)	0.0044 (17)
C17	0.083 (4)	0.031 (2)	0.044 (2)	0.011 (2)	0.014 (2)	0.0134 (19)
C18	0.071 (3)	0.034 (2)	0.037 (2)	0.019 (2)	0.004 (2)	0.0076 (18)
C19	0.056 (3)	0.037 (2)	0.039 (2)	0.014 (2)	−0.001 (2)	0.0048 (18)
C20	0.048 (2)	0.037 (2)	0.0338 (19)	−0.0006 (19)	0.0047 (18)	0.0053 (17)
C21	0.080 (4)	0.053 (3)	0.040 (2)	0.031 (3)	0.002 (2)	0.006 (2)
C22	0.097 (4)	0.047 (3)	0.034 (2)	0.034 (3)	0.006 (2)	0.0054 (19)
C23	0.100 (5)	0.057 (4)	0.042 (2)	0.032 (3)	0.003 (3)	0.010 (2)
C24	0.104 (5)	0.039 (3)	0.030 (2)	0.024 (3)	−0.008 (2)	−0.0048 (19)
C25	0.106 (5)	0.090 (5)	0.042 (3)	0.058 (4)	0.011 (3)	0.017 (3)
C26	0.116 (5)	0.056 (3)	0.037 (2)	0.040 (4)	0.003 (3)	0.007 (2)
C27	0.118 (5)	0.035 (3)	0.043 (2)	0.001 (3)	−0.048 (3)	0.000 (2)
C28	0.109 (5)	0.043 (3)	0.088 (4)	−0.025 (3)	−0.058 (4)	0.027 (3)
C29	0.097 (4)	0.034 (3)	0.063 (3)	−0.003 (3)	−0.040 (3)	0.013 (2)
Cl2	0.1117 (13)	0.0730 (10)	0.0952 (11)	0.0367 (10)	0.0665 (10)	0.0504 (9)
O12	0.0432 (15)	0.0285 (14)	0.0280 (12)	−0.0027 (12)	−0.0013 (11)	−0.0005 (11)
O13	0.0479 (16)	0.0391 (17)	0.0278 (12)	0.0071 (13)	−0.0025 (12)	−0.0065 (11)
O14	0.065 (2)	0.056 (2)	0.0382 (16)	−0.0074 (18)	−0.0110 (15)	0.0066 (15)
O15	0.0353 (13)	0.0315 (15)	0.0284 (12)	0.0013 (12)	0.0056 (11)	0.0032 (11)
O16	0.0458 (17)	0.054 (2)	0.0513 (18)	0.0085 (16)	0.0210 (15)	0.0106 (16)
O17	0.0340 (13)	0.0320 (15)	0.0307 (12)	−0.0031 (12)	0.0074 (11)	−0.0064 (11)
O18	0.071 (3)	0.095 (4)	0.140 (4)	−0.051 (3)	0.065 (3)	−0.078 (3)
O19	0.0353 (13)	0.0428 (17)	0.0278 (12)	−0.0020 (12)	−0.0020 (11)	0.0040 (12)
O20	0.0334 (15)	0.055 (2)	0.0546 (18)	0.0000 (14)	0.0025 (14)	0.0123 (16)
O21	0.0443 (16)	0.0315 (15)	0.0363 (14)	−0.0001 (13)	0.0021 (12)	0.0041 (12)
O22	0.081 (3)	0.105 (4)	0.070 (3)	−0.039 (3)	−0.014 (2)	0.035 (3)
C30	0.0300 (18)	0.034 (2)	0.0278 (16)	0.0022 (16)	0.0005 (14)	−0.0003 (15)
C31	0.039 (2)	0.031 (2)	0.0266 (16)	0.0005 (16)	−0.0004 (15)	0.0049 (15)
C32	0.0316 (17)	0.0279 (19)	0.0259 (15)	0.0009 (15)	0.0035 (14)	−0.0023 (14)
C33	0.039 (2)	0.035 (2)	0.0252 (16)	−0.0029 (17)	−0.0026 (15)	0.0026 (15)
C34	0.040 (2)	0.029 (2)	0.0269 (16)	0.0026 (16)	0.0004 (15)	0.0007 (14)
C35	0.0347 (19)	0.040 (2)	0.0278 (16)	−0.0048 (17)	0.0029 (15)	−0.0062 (16)
C36	0.039 (2)	0.055 (3)	0.0303 (18)	0.000 (2)	−0.0059 (17)	−0.0022 (19)
C37	0.055 (3)	0.101 (5)	0.034 (2)	0.011 (3)	−0.001 (2)	−0.022 (3)
C38	0.0317 (19)	0.039 (2)	0.0315 (18)	−0.0054 (17)	0.0034 (16)	0.0036 (16)
C39	0.042 (2)	0.044 (3)	0.041 (2)	0.000 (2)	0.0055 (18)	0.0118 (19)
C40	0.042 (2)	0.038 (2)	0.047 (2)	−0.0038 (19)	0.0080 (19)	−0.0136 (19)
C41	0.039 (2)	0.050 (3)	0.051 (2)	0.004 (2)	−0.001 (2)	−0.019 (2)
C42	0.037 (2)	0.036 (2)	0.045 (2)	0.0028 (18)	−0.0136 (18)	0.0097 (18)
C43	0.057 (3)	0.080 (4)	0.046 (2)	−0.013 (3)	−0.019 (2)	0.010 (3)
C44	0.050 (2)	0.0239 (19)	0.0325 (17)	−0.0057 (18)	−0.0028 (17)	−0.0028 (15)
C45	0.055 (3)	0.033 (2)	0.041 (2)	−0.005 (2)	−0.012 (2)	0.0057 (17)
C46	0.065 (3)	0.033 (2)	0.0353 (19)	−0.009 (2)	−0.009 (2)	0.0085 (17)
C47	0.062 (3)	0.038 (2)	0.035 (2)	−0.019 (2)	−0.007 (2)	0.0065 (17)
C48	0.048 (2)	0.035 (2)	0.042 (2)	−0.009 (2)	−0.0052 (19)	0.0081 (18)
C49	0.055 (2)	0.037 (2)	0.0264 (17)	−0.0087 (19)	−0.0063 (17)	0.0057 (16)
C50	0.067 (3)	0.046 (3)	0.040 (2)	−0.024 (2)	−0.006 (2)	0.008 (2)
C51	0.087 (4)	0.042 (3)	0.033 (2)	−0.025 (3)	−0.002 (2)	0.0037 (19)
C52	0.078 (4)	0.064 (4)	0.039 (2)	−0.030 (3)	−0.007 (2)	0.010 (2)
C53	0.096 (4)	0.046 (3)	0.029 (2)	−0.026 (3)	−0.001 (2)	−0.0036 (19)
C54	0.080 (4)	0.043 (3)	0.059 (3)	0.008 (3)	0.029 (3)	0.017 (2)
C55	0.099 (5)	0.061 (4)	0.075 (4)	0.031 (3)	0.049 (3)	0.035 (3)
C56	0.107 (5)	0.031 (3)	0.055 (3)	0.007 (3)	0.044 (3)	0.010 (2)
C57	0.096 (5)	0.066 (4)	0.045 (3)	−0.036 (4)	0.002 (3)	0.014 (3)
C58	0.094 (4)	0.080 (4)	0.044 (3)	−0.046 (4)	−0.005 (3)	0.015 (3)

### Geometric parameters (Å, °)

**Table:**

Cl1—C27	1.753 (5)	Cl2—C56	1.743 (5)
O1—C5	1.415 (4)	O12—C34	1.423 (4)
O1—C1	1.443 (5)	O12—C30	1.431 (5)
O2—C7	1.336 (5)	O13—C36	1.348 (5)
O2—C6	1.430 (5)	O13—C35	1.438 (5)
O3—C7	1.219 (6)	O14—C36	1.211 (6)
O4—C9	1.367 (4)	O15—C38	1.367 (4)
O4—C2	1.448 (5)	O15—C31	1.441 (4)
O5—C9	1.208 (5)	O16—C38	1.199 (5)
O6—C11	1.363 (5)	O17—C40	1.342 (5)
O6—C3	1.441 (4)	O17—C32	1.452 (4)
O7—C11	1.203 (5)	O18—C40	1.164 (5)
O8—C13	1.373 (5)	O19—C42	1.373 (5)
O8—C4	1.439 (4)	O19—C33	1.440 (4)
O9—C13	1.208 (5)	O20—C42	1.201 (5)
O10—C15	1.393 (5)	O21—C44	1.396 (5)
O10—C5	1.416 (5)	O21—C34	1.397 (4)
O11—C23	1.234 (8)	O22—C52	1.232 (7)
C1—C2	1.509 (5)	C30—C35	1.500 (5)
C1—C6	1.516 (5)	C30—C31	1.524 (5)
C1—H1	1.0000	C30—H30	1.0000
C2—C3	1.521 (5)	C31—C32	1.521 (5)
C2—H2	1.0000	C31—H31	1.0000
C3—C4	1.524 (5)	C32—C33	1.523 (5)
C3—H3	1.0000	C32—H32	1.0000
C4—C5	1.527 (5)	C33—C34	1.527 (5)
C4—H4	1.0000	C33—H33	1.0000
C5—H5	1.0000	C34—H34	1.0000
C6—H6A	0.9900	C35—H35A	0.9900
C6—H6B	0.9900	C35—H35B	0.9900
C7—C8	1.468 (7)	C36—C37	1.485 (6)
C8—H8A	0.9800	C37—H37A	0.9800
C8—H8B	0.9800	C37—H37B	0.9800
C8—H8C	0.9800	C37—H37C	0.9800
C9—C10	1.465 (6)	C38—C39	1.476 (6)
C10—H10A	0.9800	C39—H39A	0.9800
C10—H10B	0.9800	C39—H39B	0.9800
C10—H10C	0.9800	C39—H39C	0.9800
C11—C12	1.487 (5)	C40—C41	1.496 (6)
C12—H12A	0.9800	C41—H41A	0.9800
C12—H12B	0.9800	C41—H41B	0.9800
C12—H12C	0.9800	C41—H41C	0.9800
C13—C14	1.485 (6)	C42—C43	1.494 (6)
C14—H14A	0.9800	C43—H43A	0.9800
C14—H14B	0.9800	C43—H43B	0.9800
C14—H14C	0.9800	C43—H43C	0.9800
C15—C16	1.382 (6)	C44—C49	1.376 (6)
C15—C20	1.389 (6)	C44—C45	1.398 (5)
C16—C17	1.390 (7)	C45—C46	1.384 (6)
C16—H16	0.9500	C45—H45	0.9500
C17—C18	1.376 (7)	C46—C47	1.388 (7)
C17—H17	0.9500	C46—H46	0.9500
C18—C19	1.388 (6)	C47—C48	1.405 (6)
C18—C21	1.470 (6)	C47—C50	1.462 (6)
C19—C20	1.388 (6)	C48—C49	1.404 (6)
C19—H19	0.9500	C48—H48	0.9500
C20—H20	0.9500	C49—H49	0.9500
C21—C22	1.321 (7)	C50—C51	1.342 (6)
C21—H21	0.9500	C50—H50	0.9500
C22—C23	1.484 (8)	C51—C52	1.474 (7)
C22—H22	0.9500	C51—H51	0.9500
C23—C24	1.506 (7)	C52—C53	1.509 (7)
C24—C29	1.374 (9)	C53—C58	1.392 (7)
C24—C25	1.375 (7)	C53—C54	1.399 (8)
C25—C26	1.380 (7)	C54—C55	1.421 (7)
C25—H25	0.9500	C54—H54	0.9500
C26—C27	1.351 (9)	C55—C56	1.400 (9)
C26—H26	0.9500	C55—H55	0.9500
C27—C28	1.396 (8)	C56—C57	1.346 (9)
C28—C29	1.415 (7)	C57—C58	1.377 (7)
C28—H28	0.9500	C57—H57	0.9500
C29—H29	0.9500	C58—H58	0.9500
			
C5—O1—C1	111.0 (3)	C34—O12—C30	112.1 (3)
C7—O2—C6	118.0 (4)	C36—O13—C35	117.6 (3)
C9—O4—C2	117.7 (3)	C38—O15—C31	116.4 (3)
C11—O6—C3	116.9 (3)	C40—O17—C32	118.4 (3)
C13—O8—C4	116.1 (3)	C42—O19—C33	116.0 (3)
C15—O10—C5	116.3 (3)	C44—O21—C34	116.8 (3)
O1—C1—C2	106.9 (3)	O12—C30—C35	109.0 (3)
O1—C1—C6	107.9 (3)	O12—C30—C31	106.5 (3)
C2—C1—C6	115.0 (3)	C35—C30—C31	114.9 (3)
O1—C1—H1	108.9	O12—C30—H30	108.8
C2—C1—H1	108.9	C35—C30—H30	108.8
C6—C1—H1	108.9	C31—C30—H30	108.8
O4—C2—C1	107.6 (3)	O15—C31—C32	109.0 (3)
O4—C2—C3	109.5 (3)	O15—C31—C30	109.6 (3)
C1—C2—C3	110.4 (3)	C32—C31—C30	109.4 (3)
O4—C2—H2	109.8	O15—C31—H31	109.6
C1—C2—H2	109.8	C32—C31—H31	109.6
C3—C2—H2	109.8	C30—C31—H31	109.6
O6—C3—C2	107.7 (3)	O17—C32—C31	106.7 (3)
O6—C3—C4	107.8 (3)	O17—C32—C33	107.3 (3)
C2—C3—C4	108.1 (3)	C31—C32—C33	109.8 (3)
O6—C3—H3	111.0	O17—C32—H32	111.0
C2—C3—H3	111.0	C31—C32—H32	111.0
C4—C3—H3	111.0	C33—C32—H32	111.0
O8—C4—C3	110.4 (3)	O19—C33—C32	109.5 (3)
O8—C4—C5	105.7 (3)	O19—C33—C34	105.9 (3)
C3—C4—C5	109.9 (3)	C32—C33—C34	110.2 (3)
O8—C4—H4	110.2	O19—C33—H33	110.4
C3—C4—H4	110.2	C32—C33—H33	110.4
C5—C4—H4	110.2	C34—C33—H33	110.4
O1—C5—O10	108.2 (3)	O21—C34—O12	107.8 (3)
O1—C5—C4	109.0 (3)	O21—C34—C33	107.8 (3)
O10—C5—C4	107.5 (3)	O12—C34—C33	109.7 (3)
O1—C5—H5	110.7	O21—C34—H34	110.5
O10—C5—H5	110.7	O12—C34—H34	110.5
C4—C5—H5	110.7	C33—C34—H34	110.5
O2—C6—C1	111.1 (3)	O13—C35—C30	111.6 (3)
O2—C6—H6A	109.4	O13—C35—H35A	109.3
C1—C6—H6A	109.4	C30—C35—H35A	109.3
O2—C6—H6B	109.4	O13—C35—H35B	109.3
C1—C6—H6B	109.4	C30—C35—H35B	109.3
H6A—C6—H6B	108.0	H35A—C35—H35B	108.0
O3—C7—O2	122.5 (4)	O14—C36—O13	124.1 (4)
O3—C7—C8	126.0 (4)	O14—C36—C37	124.4 (4)
O2—C7—C8	111.4 (5)	O13—C36—C37	111.4 (4)
C7—C8—H8A	109.5	C36—C37—H37A	109.5
C7—C8—H8B	109.5	C36—C37—H37B	109.5
H8A—C8—H8B	109.5	H37A—C37—H37B	109.5
C7—C8—H8C	109.5	C36—C37—H37C	109.5
H8A—C8—H8C	109.5	H37A—C37—H37C	109.5
H8B—C8—H8C	109.5	H37B—C37—H37C	109.5
O5—C9—O4	121.9 (4)	O16—C38—O15	122.4 (4)
O5—C9—C10	127.7 (4)	O16—C38—C39	127.0 (4)
O4—C9—C10	110.4 (3)	O15—C38—C39	110.6 (3)
C9—C10—H10A	109.5	C38—C39—H39A	109.5
C9—C10—H10B	109.5	C38—C39—H39B	109.5
H10A—C10—H10B	109.5	H39A—C39—H39B	109.5
C9—C10—H10C	109.5	C38—C39—H39C	109.5
H10A—C10—H10C	109.5	H39A—C39—H39C	109.5
H10B—C10—H10C	109.5	H39B—C39—H39C	109.5
O7—C11—O6	123.4 (4)	O18—C40—O17	122.9 (4)
O7—C11—C12	125.7 (4)	O18—C40—C41	127.1 (4)
O6—C11—C12	110.8 (4)	O17—C40—C41	110.0 (4)
C11—C12—H12A	109.5	C40—C41—H41A	109.5
C11—C12—H12B	109.5	C40—C41—H41B	109.5
H12A—C12—H12B	109.5	H41A—C41—H41B	109.5
C11—C12—H12C	109.5	C40—C41—H41C	109.5
H12A—C12—H12C	109.5	H41A—C41—H41C	109.5
H12B—C12—H12C	109.5	H41B—C41—H41C	109.5
O9—C13—O8	121.1 (4)	O20—C42—O19	122.6 (4)
O9—C13—C14	127.2 (4)	O20—C42—C43	127.2 (4)
O8—C13—C14	111.7 (3)	O19—C42—C43	110.2 (4)
C13—C14—H14A	109.5	C42—C43—H43A	109.5
C13—C14—H14B	109.5	C42—C43—H43B	109.5
H14A—C14—H14B	109.5	H43A—C43—H43B	109.5
C13—C14—H14C	109.5	C42—C43—H43C	109.5
H14A—C14—H14C	109.5	H43A—C43—H43C	109.5
H14B—C14—H14C	109.5	H43B—C43—H43C	109.5
C16—C15—C20	121.4 (4)	C49—C44—O21	122.4 (4)
C16—C15—O10	115.8 (4)	C49—C44—C45	121.8 (4)
C20—C15—O10	122.8 (4)	O21—C44—C45	115.8 (4)
C15—C16—C17	118.8 (5)	C46—C45—C44	118.6 (4)
C15—C16—H16	120.6	C46—C45—H45	120.7
C17—C16—H16	120.6	C44—C45—H45	120.7
C18—C17—C16	121.4 (4)	C45—C46—C47	121.9 (4)
C18—C17—H17	119.3	C45—C46—H46	119.1
C16—C17—H17	119.3	C47—C46—H46	119.1
C17—C18—C19	118.5 (4)	C46—C47—C48	118.0 (4)
C17—C18—C21	123.5 (4)	C46—C47—C50	123.7 (4)
C19—C18—C21	118.0 (5)	C48—C47—C50	118.3 (5)
C18—C19—C20	121.9 (5)	C49—C48—C47	121.3 (4)
C18—C19—H19	119.1	C49—C48—H48	119.4
C20—C19—H19	119.1	C47—C48—H48	119.4
C19—C20—C15	117.9 (4)	C44—C49—C48	118.3 (4)
C19—C20—H20	121.0	C44—C49—H49	120.8
C15—C20—H20	121.0	C48—C49—H49	120.8
C22—C21—C18	127.7 (6)	C51—C50—C47	127.7 (5)
C22—C21—H21	116.1	C51—C50—H50	116.2
C18—C21—H21	116.1	C47—C50—H50	116.2
C21—C22—C23	120.4 (6)	C50—C51—C52	120.4 (5)
C21—C22—H22	119.8	C50—C51—H51	119.8
C23—C22—H22	119.8	C52—C51—H51	119.8
O11—C23—C22	121.7 (5)	O22—C52—C51	121.7 (5)
O11—C23—C24	118.9 (5)	O22—C52—C53	119.2 (5)
C22—C23—C24	119.4 (6)	C51—C52—C53	119.1 (5)
C29—C24—C25	118.8 (5)	C58—C53—C54	119.5 (5)
C29—C24—C23	122.4 (5)	C58—C53—C52	118.7 (6)
C25—C24—C23	118.8 (6)	C54—C53—C52	121.7 (5)
C24—C25—C26	122.1 (7)	C53—C54—C55	120.3 (5)
C24—C25—H25	119.0	C53—C54—H54	119.9
C26—C25—H25	119.0	C55—C54—H54	119.9
C27—C26—C25	118.3 (6)	C56—C55—C54	117.0 (6)
C27—C26—H26	120.8	C56—C55—H55	121.5
C25—C26—H26	120.8	C54—C55—H55	121.5
C26—C27—C28	123.0 (5)	C57—C56—C55	122.4 (5)
C26—C27—Cl1	119.7 (4)	C57—C56—Cl2	120.2 (5)
C28—C27—Cl1	117.2 (5)	C55—C56—Cl2	117.4 (5)
C27—C28—C29	116.7 (7)	C56—C57—C58	120.9 (6)
C27—C28—H28	121.7	C56—C57—H57	119.6
C29—C28—H28	121.7	C58—C57—H57	119.6
C24—C29—C28	121.0 (5)	C57—C58—C53	119.9 (6)
C24—C29—H29	119.5	C57—C58—H58	120.1
C28—C29—H29	119.5	C53—C58—H58	120.1
			
C5—O1—C1—C2	66.7 (3)	C34—O12—C30—C35	−168.3 (3)
C5—O1—C1—C6	−169.0 (3)	C34—O12—C30—C31	67.3 (4)
C9—O4—C2—C1	−133.4 (3)	C38—O15—C31—C32	95.5 (4)
C9—O4—C2—C3	106.6 (4)	C38—O15—C31—C30	−144.8 (3)
O1—C1—C2—O4	178.9 (3)	O12—C30—C31—O15	178.5 (3)
C6—C1—C2—O4	59.1 (4)	C35—C30—C31—O15	57.8 (4)
O1—C1—C2—C3	−61.6 (4)	O12—C30—C31—C32	−62.0 (4)
C6—C1—C2—C3	178.5 (3)	C35—C30—C31—C32	177.2 (3)
C11—O6—C3—C2	−148.9 (3)	C40—O17—C32—C31	−127.7 (4)
C11—O6—C3—C4	94.7 (4)	C40—O17—C32—C33	114.7 (4)
O4—C2—C3—O6	58.6 (4)	O15—C31—C32—O17	60.0 (4)
C1—C2—C3—O6	−59.7 (4)	C30—C31—C32—O17	−59.9 (4)
O4—C2—C3—C4	174.8 (3)	O15—C31—C32—C33	175.9 (3)
C1—C2—C3—C4	56.5 (4)	C30—C31—C32—C33	56.1 (4)
C13—O8—C4—C3	−77.2 (4)	C42—O19—C33—C32	−71.9 (4)
C13—O8—C4—C5	163.9 (3)	C42—O19—C33—C34	169.3 (3)
O6—C3—C4—O8	−53.8 (4)	O17—C32—C33—O19	−52.2 (4)
C2—C3—C4—O8	−170.0 (3)	C31—C32—C33—O19	−167.8 (3)
O6—C3—C4—C5	62.4 (4)	O17—C32—C33—C34	64.0 (4)
C2—C3—C4—C5	−53.7 (4)	C31—C32—C33—C34	−51.6 (4)
C1—O1—C5—O10	178.0 (3)	C44—O21—C34—O12	−85.7 (4)
C1—O1—C5—C4	−65.5 (4)	C44—O21—C34—C33	156.0 (3)
C15—O10—C5—O1	−88.4 (4)	C30—O12—C34—O21	178.8 (3)
C15—O10—C5—C4	154.1 (3)	C30—O12—C34—C33	−64.1 (4)
O8—C4—C5—O1	177.4 (3)	O19—C33—C34—O21	−70.0 (4)
C3—C4—C5—O1	58.3 (4)	C32—C33—C34—O21	171.6 (3)
O8—C4—C5—O10	−65.5 (4)	O19—C33—C34—O12	172.8 (3)
C3—C4—C5—O10	175.4 (3)	C32—C33—C34—O12	54.4 (4)
C7—O2—C6—C1	−119.7 (4)	C36—O13—C35—C30	−115.0 (4)
O1—C1—C6—O2	−62.2 (4)	O12—C30—C35—O13	−66.4 (4)
C2—C1—C6—O2	57.0 (5)	C31—C30—C35—O13	52.9 (5)
C6—O2—C7—O3	3.6 (6)	C35—O13—C36—O14	5.4 (6)
C6—O2—C7—C8	−174.2 (4)	C35—O13—C36—C37	−172.4 (4)
C2—O4—C9—O5	−1.5 (6)	C31—O15—C38—O16	−0.2 (5)
C2—O4—C9—C10	177.4 (3)	C31—O15—C38—C39	−177.7 (3)
C3—O6—C11—O7	4.0 (6)	C32—O17—C40—O18	−0.3 (7)
C3—O6—C11—C12	−173.8 (3)	C32—O17—C40—C41	−177.5 (3)
C4—O8—C13—O9	−2.7 (6)	C33—O19—C42—O20	−6.4 (6)
C4—O8—C13—C14	177.2 (3)	C33—O19—C42—C43	170.7 (4)
C5—O10—C15—C16	−151.4 (4)	C34—O21—C44—C49	23.5 (5)
C5—O10—C15—C20	28.1 (5)	C34—O21—C44—C45	−155.7 (3)
C20—C15—C16—C17	−3.2 (7)	C49—C44—C45—C46	−2.4 (6)
O10—C15—C16—C17	176.3 (4)	O21—C44—C45—C46	176.8 (3)
C15—C16—C17—C18	0.4 (7)	C44—C45—C46—C47	−0.7 (7)
C16—C17—C18—C19	2.1 (7)	C45—C46—C47—C48	2.5 (7)
C16—C17—C18—C21	178.3 (5)	C45—C46—C47—C50	−179.2 (4)
C17—C18—C19—C20	−1.9 (7)	C46—C47—C48—C49	−1.2 (6)
C21—C18—C19—C20	−178.2 (4)	C50—C47—C48—C49	−179.7 (4)
C18—C19—C20—C15	−0.8 (7)	O21—C44—C49—C48	−175.6 (4)
C16—C15—C20—C19	3.4 (6)	C45—C44—C49—C48	3.5 (6)
O10—C15—C20—C19	−176.1 (4)	C47—C48—C49—C44	−1.7 (6)
C17—C18—C21—C22	−2.4 (8)	C46—C47—C50—C51	−2.5 (8)
C19—C18—C21—C22	173.8 (5)	C48—C47—C50—C51	175.9 (5)
C18—C21—C22—C23	−178.9 (5)	C47—C50—C51—C52	−176.7 (4)
C21—C22—C23—O11	4.5 (9)	C50—C51—C52—O22	4.8 (8)
C21—C22—C23—C24	−176.6 (5)	C50—C51—C52—C53	−176.7 (4)
O11—C23—C24—C29	−171.2 (6)	O22—C52—C53—C58	7.6 (8)
C22—C23—C24—C29	9.9 (8)	C51—C52—C53—C58	−170.9 (5)
O11—C23—C24—C25	7.6 (8)	O22—C52—C53—C54	−169.1 (6)
C22—C23—C24—C25	−171.3 (5)	C51—C52—C53—C54	12.4 (7)
C29—C24—C25—C26	1.3 (9)	C58—C53—C54—C55	−0.6 (8)
C23—C24—C25—C26	−177.6 (5)	C52—C53—C54—C55	176.1 (5)
C24—C25—C26—C27	−0.1 (9)	C53—C54—C55—C56	1.7 (9)
C25—C26—C27—C28	0.6 (9)	C54—C55—C56—C57	−1.2 (9)
C25—C26—C27—Cl1	177.5 (4)	C54—C55—C56—Cl2	−179.4 (4)
C26—C27—C28—C29	−2.2 (8)	C55—C56—C57—C58	−0.4 (10)
Cl1—C27—C28—C29	−179.1 (4)	Cl2—C56—C57—C58	177.7 (5)
C25—C24—C29—C28	−2.9 (8)	C56—C57—C58—C53	1.5 (9)
C23—C24—C29—C28	175.9 (5)	C54—C53—C58—C57	−1.0 (9)
C27—C28—C29—C24	3.3 (8)	C52—C53—C58—C57	−177.8 (5)

### Hydrogen-bond geometry (Å, °)

**Table:**

*D*—H···*A*	*D*—H	H···*A*	*D*···*A*	*D*—H···*A*
C1—H1···O9^i^	1.00	2.38	3.185 (5)	137
C20—H20···O1	0.95	2.54	3.111 (5)	118
C20—H20···O9^i^	0.95	2.54	3.271 (5)	134
C30—H30···O20^i^	1.00	2.51	3.316 (5)	138
C32—H32···O20	1.00	2.53	3.031 (5)	111
C49—H49···O12	0.95	2.50	3.065 (5)	119
C49—H49···O20^i^	0.95	2.53	3.271 (6)	135

Symmetry codes: (i) *x*−1, *y*, *z*.
